# 

**Published:** 1868-09

**Authors:** 


### Books and Pamphlets Received.

The Science and Practice of Medicine. By William Aitkins, M. D., Edinburgh, Professor of Pathology in the Army Medical School. Second American, from the fifth, enlarged and carefully revised London edition, adopting new nomenclature of the Royal College of Physicians of London, with large editions. By Meredith Clymer, M. D., ex-Professor of the institutes and practice of medicine in the University of New York; formerly Physician to the Philadelphia Hospital, etc., etc., in two volumes, with a map, lithographic plates, and numerous illustrations. Vol. 1. Philadelphia: Lindsay & Blakiston, 1868.

A Theoretical and Practical Treatise on Midwifery, including the diseases of Pregnancy and Parturition. By P. Cazeaux, member of the Imperial Academy of Medicine; adjunct Professor in the Faculty of Medicine of Paris; Chevalier of the Legions of Honor; correspondent of the Society of Accoucheurs of Berlin; President of the Medical Society of the Seine, etc., etc. Adopted by the superior council of public instructions, and placed by ministerial decision, in the ranks of classical works, designed for the use of midwife students, in the Maternity Hospital of Paris. Revised and annotated by Wm. R. Bullock, M. D., with one hundred and seventy-five illustrations. Philadelphia: Lindsay & Blakiston, 1868.

On Diseases peculiar to Women, including displacements of the Uterus. By Hugh L. Hodge, M. D., Emeritus Professor of Obstetrics and Diseases of Women and Children in the University of Pennsylvania, with illustrations. Second edition, revised and enlarged. Philadelphia: Henry C. Lea, 1868.

Diseases of Children. A Clinical Treatise based on Lectures delivered at the Hospital for Sick Children, London. By Thomas Hillier, M. D., London, Fellow of the Royal College of Physicians, Physician to the Hospital for Sick Children, and to University College Hospital, London. Philadelphia: Lindsay & Blakiston, 1868.

Criminal Abortion; its Nature, its evidence, and its Law. By Horatio R. Storer, M. D., L. L. B., Fellow of the American Academy of Arts and Sciences, and late Professor of Obstetrics and Medical Jurisprudence in Berkshire Medical College; and Franklin Fiske Heard. Boston: Little, Brown & Co., 1868.

Vesico-Vaginal Fistula from parturition and other causes; with cases of Recto-Vaginal Fistula. By Thomas Addis Emmett, M. D., Surgeon-in-Chief of the New York State Women's Hospital, etc., etc. New York: Wm. Wood & Co., 1868.

A Manual on Extracting Teeth, founded on the Anatomy of the parts involved in the operation; the kinds and proper construction of the instruments to be used; the accidents liable to occur from operation, and the proper remedies to retrieve such accidents. By Abraham Robertson, D. D. S., M. D. Second edition. Philadelphia: Lindsay & Blakiston, 1868.

The Physician's Visiting List for 1869. Eighteenth year of its publication. Philadelphia: Lindsay & Blakiston.

Ovariotomy: a paper read before the Ohio State Medical Society, at its annual meeting held at Delaware, June, 1868. By Alexander Dunlap, A. M., M. D., of Springfield, Ohio.

Transactions of the Fifteenth Annual Meeting of the Medical Society of the State of North Carolina, held at Warranton, N. C., 20th May, 1868.

Announcement of the Philadelphia School of Anatomy. Winter session of 1868-9.

Announcement of the Medical Department of the Cumberland University, Nashville, Tenn., 1868.

Dr. Walters' Doctrines of Life. Reply to the letter of Dr. Carpenter.

Indiana State Medical Society, 1868. Report of Cholera, by Geo. Sutton, M. D., Aurora, Ind.

Transactions of the Indiana State Medical Society at its eighteenth annual session, held at Indianapolis, May 19th and 20th, 1868.

Illustrated Catalogue of Medical, Surgical and Scientific Publication, 1868. Philadelphia: Henry C. Lea, 1868.

Transactions of the Medical Society of the State of Pennsylvania, at its nineteenth annual session, held at Harrisburg, June, 1868.

Progressive Locomotor Ataxia. By Walter Coles, M. D., Professor of Diseases of Women and Children, Medical College of Virginia.

